# The Physician’s Visiting List

**Published:** 1891-01

**Authors:** 


THE PHYSICIAN'S VISITING LIST. Philadelphia: P. Blakiston, Son & Co., 1012 Walnut St.

This popular visiting list, now in the fortieth year of its publication, is replete with useful information for the busy practitioner. It can be easily carried in the pocket, is pliable, and is one of the necessities which a physician cannot dispense with.

